# Autologous tumor cell vaccination combined with systemic CpG-B and IFN-α promotes immune activation and induces clinical responses in patients with metastatic renal cell carcinoma: a phase II trial

**DOI:** 10.1007/s00262-019-02320-0

**Published:** 2019-03-09

**Authors:** Bas D. Koster, Saskia J. A. M. Santegoets, Jorien Harting, Arnold Baars, S. Marieke van Ham, Rik J. Scheper, Erik Hooijberg, Tanja D. de Gruijl, Alfons J. M. van den Eertwegh

**Affiliations:** 1 Departments of Medical Oncology, Amsterdam UMC, Vrije Universiteit, Cancer Center Amsterdam, De Boelelaan 1117, 1081 HV Amsterdam, The Netherlands; 2 Department of Medical Oncology, Leiden University Medical Center, Hippocratespad 21, 2333 ZD Leiden, The Netherlands; 3 Departments of Pathology, Cancer Center Amsterdam, Amsterdam UMC, Vrije Universiteit, De Boelelaan 1117, 1081 HV Amsterdam, The Netherlands; 4 Department of Immunopathology, Landsteiner Laboratory, Amsterdam UMC and Swammerdam Institute for Life Sciences, Sanquin Research, University of Amsterdam, Meibergdreef 9, 1105 AZ Amsterdam, The Netherlands; 5 Department of Pathology, Antoni van Leeuwenhoek/Netherlands Cancer Institute, Plesmanlaan 121, 1066 CX Amsterdam, The Netherlands

## Introduction

Up until the last decade, the treatment options for metastatic renal cell carcinoma (mRCC) patients were limited. mRCC is resistant to systemic cytotoxic chemotherapy [1] and cytokine-based therapies like IFN-α and IL-2 resulted in modest response rates and little survival benefit [2]. Over the past decade, the treatment of mRCC has changed considerably with the introduction of targeted therapies and, more recently, immune checkpoint inhibitors (ICI) [3]. Although the introduction of targeted therapies has markedly improved patient outcome, they rarely induce complete responses, and most patients eventually develop resistance to these therapies. Clinical trials with ICI nivolumab (anti PD-1) and ipilimumab (anti CTLA-4) in mRCC reconfirmed the relative tractability of this tumor type to immunotherapy. However, the objective response rate of mRCC patients who received combination treatment of nivolumab and ipilimumab is still only 42% and comes at the cost of substantial (although often manageable) toxicity [4]. Therefore, further exploration of immunotherapeutic combination approaches is warranted for the treatment of mRCC.

Recent insights have linked responses to immune checkpoint blockade to mutation burden and the frequency of neo-antigens [5]. Vaccines aimed at priming or boosting T cell responses to neoantigens may thus increase response rates to ICI [6]. Unfortunately, the highly individualized nature of these neoantigens makes them hard to leverage through therapeutic vaccination. Autologous tumor cell vaccination (ATV) is a strategy to induce a specific immune response against tumor cells and their particular antigens, including neoantigens, without the need for prior identification of actionable T cell epitopes. Whole tumor cell vaccines have shown clinical and immunological activity in mRCC patients [7–9], as well as in patients with other tumor types [10–12]. To increase the immune response against autologous tumor cells (ATC), the whole cell vaccine can be combined with adjuvants. We have demonstrated in the past that ATV and BCG prolonged disease free survival in stage-II colorectal cancer and improved survival in stage-III/IV melanoma patients, which correlated significantly with a positive post-vaccination DTH response [13, 14]. Unfortunately, BCG is relatively toxic as it can cause ulcerations [13–15]. The discovery that unmethylated cytosine-phosphate-guanine oligodeoxynucleotides (CpG ODN) are the active elements in bacterial DNA and can directly activate and induce maturation of B cells and plasmacytoid dendritic cells (pDC) has led to the development of CpG ODN as treatment modality and vaccine adjuvant for infectious diseases and cancer [16, 17]. Indeed, B-class CpG ODN (CpG-B) has been demonstrated to enhance vaccine responses to hepatitis B, malaria and cancer [18–23].

We conducted a phase II clinical trial with the primary objective of investigating whether the treatment with ATV, CpG-B and IFN-α was feasible and tolerable and resulted in higher clinical response rates than IFN-α alone (by historical controls). Secondary objectives were to assess progression-free survival and overall survival of treated patients compared to historical data. Here, we report on the biological and clinical efficacy of this experimental treatment.

## Materials and methods

### Patients

Patients with bi-dimensional measurable metastases of histologically proven RCC, and in whom progression before or after nephrectomy had been demonstrated, were eligible for this trial. Furthermore, a WHO performance status of 0 or 1 was required and patients were only eligible when sufficient numbers of tumor cells were available for the production of a minimum of three vaccines. Patients with a history of autoimmune- or antibody-associated disease, prior malignancy, patients who were using immune suppressive drugs, or who had undergone prior immunotherapy for metastatic disease (e.g., IL-2 or IFN-α treatment) were excluded.

**Fig. 1:**
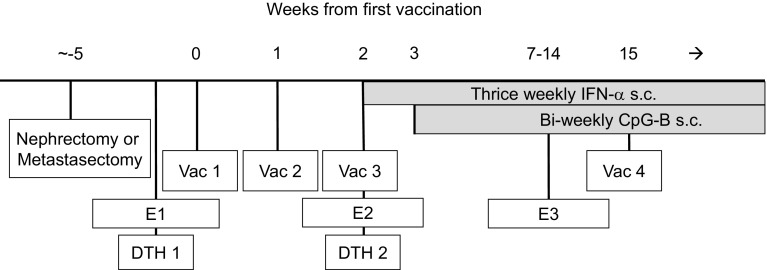
ATV treatment scheme. *E1, E2, E3* Evaluation 1, 2 and 3 (i.e., time of heparinized blood collection), *DTH* delayed type hypersensitivity, *Vac 1, Vac 2, Vac 3* vaccination 1, 2 and 3, *IFN-α* interferon alpha, *s.c*. subcutaneous, *CpG-B* cytosine-phosphate-guanine Class B

During the first month of therapy, the patients were seen bi-weekly. Thereafter, follow-up visits started at E3 (see Fig. 1) and were scheduled every 12 weeks or at treatment discontinuation due to disease progression. At each follow-up visit, the patients were subjected to a physical examination including WHO performance status, blood panels and a tumor measurement to define response which was assessed on the basis of a set of “target lesions” chosen before the first vaccination. Response (at E3) was defined with computed tomography (CT) scans according to the WHO criteria for response.

The primary endpoint of the study was tumor response compared to historical data. Secondary endpoints included toxicity, progression-free survival, overall survival, the relation between DTH responses against tumor cells and clinical responses, and the value of pre-vaccination tumor-specific T cell reactivity as a predictor of successful immunotherapy.

Of note, IFN-α was the only treatment option for potentially eligible patients at the start of this trial. However, the enrolment of this study was halted in 2006 when sunitinib became available. Therefore, enrolment stopped at 15 patients instead of 41 as originally planned.

### Vaccine preparation

Patients underwent a total nephrectomy or, if the kidney was already removed before inclusion, a metastasectomy. The tissue that was not used for pathologic diagnosis and staging was transferred to our vaccine production laboratory within 48 h of the surgery. The tumor tissue was then dissociated as previously described [13, 24]. Briefly, tumor tissue was cut into small pieces and subsequently incubated in a 0.1% DNase I, 0.14% collagenase (Boehringer) solution. After 45 min incubation at 37 °C, single cells were harvested and remaining tumor fragments again suspended in a DNase/collagenase solution; this cycle was repeated 3–4 times, after which single cells were harvested through a 100 µm gauze, a sample for bacteriology control was taken and viability was tested using trypan blue exclusion. Cells were aliquoted (at 15–20 × 10^6^ viable cells per vial) and cryopreserved using a linear freezer. Vials were stored in liquid nitrogen until vaccination. Prior to vaccination, the frozen tumor cells were irradiated (20,000 rad), thawed, counted and assessed for viability. For each patient, we aimed to produce as many vaccines as possible.

### Vaccination procedure

Vaccination started 4–6 weeks after nephrectomy or metastasectomy. For every vaccine 0.7–1.3 ×10^7^ viable autologous tumor, cells were used and 100 µg GM-CSF (Leukine; Berlex Laboratories Inc.), 1 mg CpG-B (CPG 7909, Coley Pharmaceutical Group, inc. Wellesley, MA 02481 USA) and 50 µg Keyhole Limpet Hemocyanine (KLH) (Calbiochem) were added. Vaccines were administered intradermally. For individual evaluation of the injection sites, different locations were used for each administration as previously described for ATV in patients with colon carcinoma [13]. The local injection site reactions were monitored and documented at each visit. All patients received 3 weekly intradermal injections of the vaccine, followed by booster vaccinations every 3 months for as long as the vaccines lasted and the disease did not progress. After the first three vaccinations, patients were treated bi-weekly with 8 mg of CpG-B s.c. and 6 MU IFN-α s.c. three times per week for at least 3 months to enhance both innate immunity and the effector phase of the specific immunity. To prevent additive toxicity and to enable a separate observation of toxicity of CpG-B and IFN-α, these two compounds were never administered on the same day. IFN-α was administered for a maximum of 1 year and CpG-B for a maximum of 2 years or until disease progression, grade III/IV toxicity, or death.

### Sampling of peripheral blood

For immune monitoring, heparinized blood samples were taken from the patients before start of therapy [evaluation 1 (E1)], at the day of the third vaccination [evaluation 2 (E2)] and 5–12 weeks after the third vaccination [evaluation 3 (E3)] (see Fig. 1 for treatment and evaluation scheme). PBMC were isolated by density centrifugation (Nycomed AS, Oslo, Norway) and subsequently cryopreserved for later analysis as previously described [25].

### Antibodies and four-color flow cytometry

Peripheral blood lymphocyte, monocyte, peripheral blood dendritic cell (PBDC) and monocytoid myeloid-derived suppressor cell (mMDSC) frequencies and activation status were assessed before and during treatment by four-color flow cytometry staining. Cell surface antibody staining of PBMC was performed in PBS/0.1% BSA/0.02% Sodium-Azide for 30 min at 4 °C. The following antibodies were used: FITC, PE, PerCP-Cy5.5 or APC-labeled Abs directed against human CD3, CD4, CD8, CD11c, CD14, CD15, CD16, CD19, CD25, CD27, CD33, CD45, CD45RO, CD45RA, CD56, CTLA4, CD123, HLA-DR, PD-1 (all BD Biosciences), CD11b, FoxP3 (eBioscience, San Diego, CA), CD40 (Beckman Coulter, Marseille, France), Fab-M-FITC (Southern Biotec, Birmingham, AL), and blood DC antigens BDCA1, BDCA2, BDCA3 (all from Milteny Biotec, Bergisch Gladbach, Germany) and MDC8 (a kind gift from Dr. E.P. Rieber, Dresden, Germany) and matching isotype control antibodies. Intracellular FoxP3 and CTLA-4 staining was conducted with the anti-human FoxP3 staining kit (eBioscience, San Diego, CA) according to the manufacturers’ protocol. Stained cells were analyzed on a FACScalibur (BD Biosciences) using Cell Quest software.

### T cell subset and differentiation state definitions

Naive CD4^+^ or CD8^+^ T cells (Tn) were defined as CD27^+^CD45RO^−^ cells, effector T cells (Teff) as CD27^−^CD45RO^+^, central-memory CD4^+^ T cells (Tcm) as CD27^+^CD45RO^+^ cells and effector-memory cells (Tem) as CD27^−^CD45RO^+^ [26]. Tregs were defined as CD3^+^CD4^+^CD25^hi^, and FoxP3^+^. As FoxP3 has also been described to be transiently up-regulated on dividing (activated) effector T cells [27–29], we also analyzed FoxP3 expression within these activated (effector-like) T cells, which we defined as CD4^+^CD25^intermediate^ (CD4^+^CD25^int^) cells. For Treg gating procedures, we refer to Huijts et al. 2017 [30].

### Myeloid subset definitions

PBDC frequencies were determined on the basis of expression of BDCA or MDC-8 markers: cDC1 was detected as CD11c^+^CD14^−^ BDCA3^+^ [31]. DC belonging to the so-called conventional DC2 (cDC2) subset was identified as: CD11c^hi^CD19^−^CD14^−^BDCA1/CD1c^+^; non-classical monocytes [32] were detected as CD11c^+^CD14^lo^MDC8^+^ (also previously known as 6-sulfo LacNAc^+^ or SLAN-DC [33, 34]) and pDC were detected as CD11c^−^CD14^−^CD123^hi^BDCA2^+^ [35]. Classical monocytes were defined as CD14^hi^ and mMDSC were defined as Lin^−^CD14^+^HLA-DR^neg/lo^ cells [36]. Activation status of the above-mentioned cDC and pDC subsets was determined by calculating the median fluorescence index (MFI) of CD40 expression by dividing the median fluorescence (med. fl.) of the CD40 antibody by the med. fl. of the isotype-control antibody. For detailed gating procedures, we refer to Santegoets et al. [37], including its supplementary materials.

### Tumor-specific T cell reactivity and IFN-γ ELISA

Tumor reactivity of T cells in peripheral blood before, during and after ATV was assessed by IFN-γ secretion. To this end, ATC suspensions were used as stimulator cells in an overnight stimulation assay. Tumor cell suspensions were thawed and resuspended in IMDM medium (Lonza, Verviers, Belgium) supplemented with 10% FCS (Hyclone, Amsterdam, The Netherlands), 100 I.E./ml sodium penicillin (Yamanouchi Pharma, Leiderdorp, The Netherlands), 100 µg/ml streptomycin sulphate (Radiumfarma-Fisiopharma, Naples, Italy), 2.0 mM l-glutamine (Invitrogen, Breda, The Netherlands) and 0.01 mM 2-mercapoethanol (Merck, Darmstadt, Germany; hereafter referred to as complete medium). Next, 50,000 ATC and 100,000 PBMC were cultured either alone or together for 20 h in complete medium in a 96-well round-bottom plate, after which supernatants were harvested and frozen. IFN-γ levels were determined by ELISA (sensitivity 1 pg/ml) according to manufacturer’s instructions (M1933, Sanquin, Amsterdam, The Netherlands). IFN-γ levels are given as the mean IFN-γ concentration in pg/ml per 1 × 10e6 PBMC/ml ± SD of triplicate wells. Responses were considered positive when the amount of IFN-γ produced by PBMC in response to ATC was at least twice the amount of the sum of IFN-γ detected in overnight unstimulated PBMC or ATC mono-cultures, and was at least 10 pg/ml. Mean ATC-specific IFN-γ concentration was calculated by subtracting the IFN-γ levels from ATC + PBMC alone from the IFN-γ levels from ATC:PBMC co-cultures.

### Delayed type hypersensitivity (DTH) response assessment

Several studies have demonstrated that the size of a DTH response after autologous tumor cell vaccination strongly correlates with recurrence and survival of cancer [7, 14, 39]. The presence of a DTH response to tumor cells is a measure of immunogenicity and reflects the efficacy of the vaccination and the general immune status of the patient. In our study, DTH skin tests were performed prior to the first vaccination and at the time of the third vaccination. To this end, 2 × 10^6^ ATC and 5 µg KLH were injected intradermally into separate sites and 48 h later the DTH response was evaluated by measuring the induration by the “Sokal pen method”[38]. In brief, a line was drawn with a pen 1–2 cm away from the margin of the skin test reaction towards the lesion. The pen was held at a 45° angle and the pen was advanced with moderate pressure until resistance was met. This procedure was repeated four times. Next, induration was measured between opposing points by centimeter ruler. Total induration was calculated as an ellipse (π*ab*) and given in mm^2^.

### Statistics

#### Sample size

The response rate for IFN-α treatment, taken from historical data, was assumed to be 10% [2]. A response rate of 25% for the combination of ATV, CpG-B and IFN-α was expected and initially planned as primary outcome of this study. To detect this increase with a two-sided test (*α* = 0.05) and 80% power, 41 evaluable patients had to be enrolled in the study. Abortion of the enrolment after 15 patients disabled us to perform a reliable response rate analysis. However, the sample size proved sufficient to obtain significance levels in the immune monitoring analyses.

#### Statistical analyses

Differences between immune parameters before (E1) and during treatment (E2 or E3) were analyzed with a two-tailed paired t test. A two-tailed unpaired t test was used for the analysis of the difference in induration area caused by ATC and KLH between patients with clinical benefit (CB) (CR, PR and SD) and patients without clinical benefit (NCB) (PD) and for the analysis of the difference in tumor-specific T cell reactivity between CB and NCB before and during treatment. Microsoft Excel (version 2010) and GraphPad Prism (Version 6.02) were used for all graphs, tables and analyses. Differences were considered significant when *p* ≤ 0.05.

## Results

### Patient characteristics

Between April 2004 and April 2006, 30 mRCC patients were assessed for eligibility to enter this single-centre, single-arm phase II trial. 90% (*n* = 27) of the patients had sufficient tumor material for vaccine preparation. 15 patients were eventually enrolled and 12 patients were excluded due to death prior to the first vaccination (*n* = 5), additional malignancies (*n* = 3), the absence of progression or metastasis (*n* = 3) or poor renal function (*n* = 1). We refer to Table 1 for detailed characteristics of the 15 enrolled patients.

**Table 1:** Patient characteristics

Patient	Age	Sex (M/F)	ECOG status	MSKCC risk factors	Histologic subtype	Metastatic sites	Response	Response (in months)
1	61	M	1	3	ccRCC	Lungs, liver, MLN, RLN	PD	–
2	74	M	0	2	ccRCC	Lungs, liver, MLN	PD	–
3	45	M	0	2	ccRCC	Lungs, MLN	SD	3.6
4	69	M	1	3	ccRCC	Liver, MLN, RLN	PD	–
5	56	M	1	2	ccRCC	Lungs, bone, MLN, RLN	PD	–
6	52	M	1	2	ccRCC	MLN	PD	–
7	54	M	0	1	ccRCC	Lungs, MLN	PR	7
8	60	F	1	4	ccRCC/ sRCC	Lungs, bone, MLN, RLN	PD	–
9	57	M	0	2	ccRCC/ sRCC	Lungs, Skin, MLN, intramuscular	PD	–
10	60	F	0	1	ccRCC	Lungs	SD	5.1
11	62	M	0	2	ccRCC	Lungs, MLN, RLN	PR	6.5
12	71	M	0	2	ccRCC	Lungs, MLN	CR	146+
13	57	F	0	2	ccRCC	Lungs, liver, MLN, RLN	PD	–
14	73	F	0	2	ccRCC	Lungs, bone, RLN	SD	4.4
15	59	M	0	3	ccRCC	Lungs, bone, MLN	PD	–

*M* Male, *F* Female, *ECOG* Eastern Cooperative Oncology Group, *MSKCC* Memorial Sloan-Kettering Cancer Center, *ccRCC* clear cell Renal Cell Carcinoma, *sRCC* sarcomatoid Renal Cell Carcinoma, *MLN* Mediastinal Lymph Node, *RLN* Retroperitoneal Lymph Node, *CR* Complete Response, *PR* Partial Response, *SD* Stable Disease, *PD* Progressive Disease

### Clinical results

**Fig. 2:**
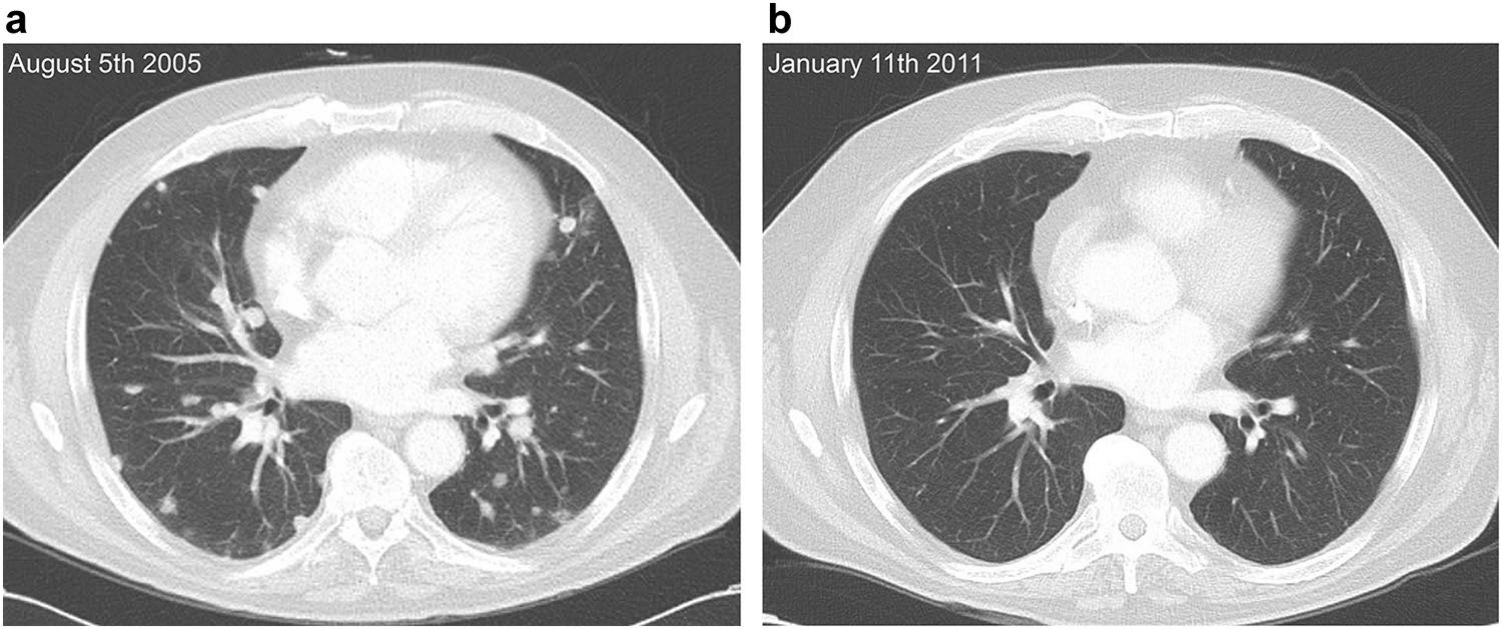
Pre-treatment lung window CT scan of the patient that had a complete response shows multiple solid nodules in both lungs (**a**). In the most recent lung window CT scan (approximately, 5 and a half years later), no solid lung nodules are identified (**b**)

All 15 patients received the first three vaccines and were, therefore, included in the current analysis. Objective clinical responses occurred in three patients, including one CR and two PR. The CR is still ongoing for more than 12 years and PR lasted 6.5 and 7 months. The patient with the CR was alive at the last moment of follow-up (October 2017) and has not shown any signs of disease since the experimental treatment and, therefore, never received any other form of therapy for RCC (See Fig. 2 for pre- and post-treatment lung window CT scans). Three patients had SD which lasted between 3.6 and 5.1 months. The six patients with CR, PR and SD were designated patients with CB. Nine patients developed PD and were designated patients with NCB (Table 1) and, therefore, did not receive any booster vaccines. From the six patients with CB, four patients received one and two patients received two booster vaccinations after which there was either no ATV available anymore or disease progression occurred.

### Adverse events

Adverse events to the experimental treatment were relatively mild with grade one and two fever and fatigue being the most common events. Elevated gamma-glutamyl transferase and alkaline phosphatase (in six and three patients, respectively) were the most prominent grade three or four adverse events that might have been related to the experimental treatment. Furthermore, two cases of grade three or four anaemia were recorded and grade three or four melena, fatigue, dizziness, disturbed balance, sensory neuropathy, aphasia, hemiplegia were all recorded once (Supplemental table 1) which were all considered to be unrelated to the experimental treatment (e.g., neurological adverse events were found in one patient who suffered from brain metastases). Common local toxicity consisted of induration and edema at the vaccination site. We did not observe any ulcerations at the vaccination sites.

### Delayed type hypersensitivity (DTH) response

**Fig. 3:**
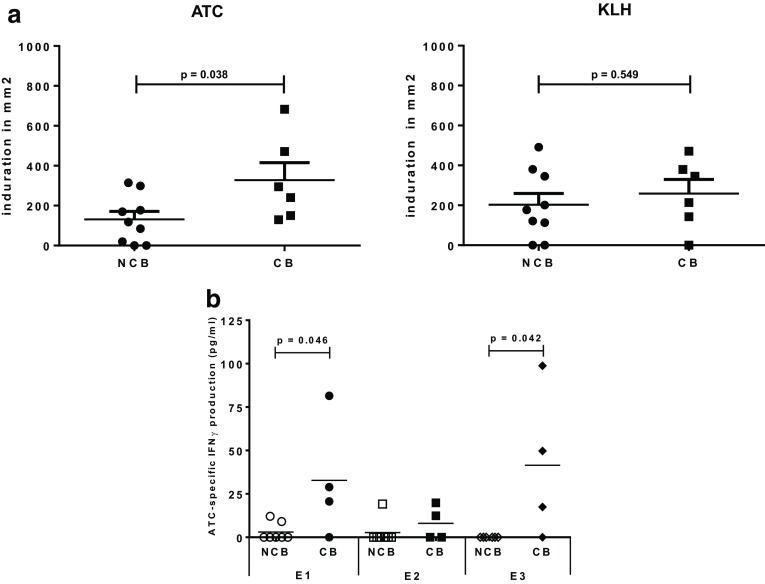
Delayed type hypersensitivity (DTH) response against autologous tumor cells (ATC) and keyhole limpet hemocyanine (KLH) at the time of the third vaccination and DTH2 (E2) in mm^2^ (**a**). ATC-specific IFN-ɣ production (pg/ml) determined by ELISA at baseline (E1), third vaccination (E2), and follow-up (7–14 weeks, E3) for patients with clinical benefit [CB: stable disease (SD), partial response (PR) and complete response (CR)] and patients with no clinical benefit [NCB: progressive disease (PD)] (**b**)

None of 15 patients demonstrated a positive skin test in response to ATC before vaccination, but 13 of them showed a positive skin test upon treatment. 12 of 15 patients demonstrated a positive skin test in response to KLH after vaccination. The median induration in response to ATC was 169.6 mm^2^ and to KLH was 201.1 mm^2^. Interestingly, we observed a significant difference in the size of the DTH response between patients with CB (i.e., stronger DTH reaction) and patients with NCB for ATC (*p* = 0.038), but not for KLH (Fig. 3a).

### Tumor-specific T cell reactivity and IFN-γ ELISA

Similarly to our findings for DTH responsiveness to ATC, ATC recognizing circulating T cells were revealed to be more frequent in patients who clinically benefitted from the therapy compared to patients with NCB (i.e.three of four in CB versus two of seven in NCB patients; *p* = 0.061 with two-sided Fisher’s exact test; data not shown). We also found that the magnitude of the IFN-γ response was significantly higher in patients with CB at E1 (*p* = 0.046) and at E3 (*p* = 0.042) but, interestingly, not at E2 (Fig. 3b).

### B and T cell activation

**Fig. 4:**
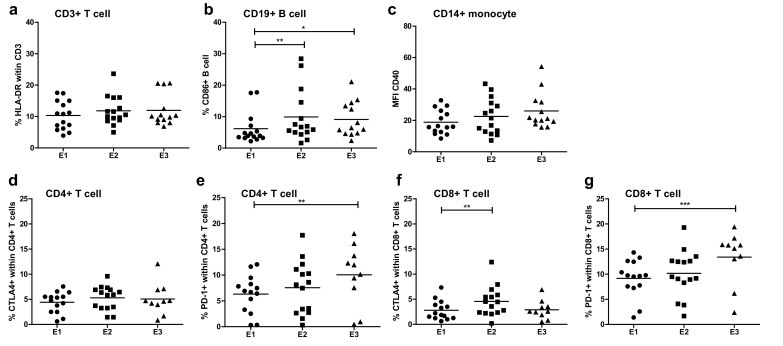
Activation markers at baseline (E1), third vaccination (E2), and follow-up (7–14 weeks, E3) on T cells (HLA-DR) (**a**), B cells (CD86) (**b**) and monocytes (CD40) (**c**). Activation markers CTLA4 and PD1 are shown separately on CD4 + T cells (**d** and **e**) and CD8 + T cells (**f** and **g**)

No significant differences were observed in overall frequencies of circulating CD3^+^ T cells, CD19^+^ B cells, CD3^−^CD56^+^ NK cells, CD14^hi^ classical monocytes and CD4^+^CD25^hi^FoxP3^+^ Tregs, nor in frequencies of circulating CD4^+^ T cells, CD8^+^ T cells and CD4^+^ or CD8^+^ T naive, Teff, Tcm or Tem cells following treatment (Supplemental figure. 1). However, ATV delivery induced activation of B cells and T cells, as reflected by significantly increased percentages of CD19^+^CD86^+^ B cells and of CD4^+^PD-1^+^, CD8^+^ PD-1^+^ and CD8^+^CTLA-4^+^ T cells following treatment (see Fig. 4). Yet, no correlations between baseline levels or increases in activated B or T cell rates and treatment response were observed (data not shown).

### Treatment-induced changes in myeloid subset frequencies and activation states

**Fig. 5:**
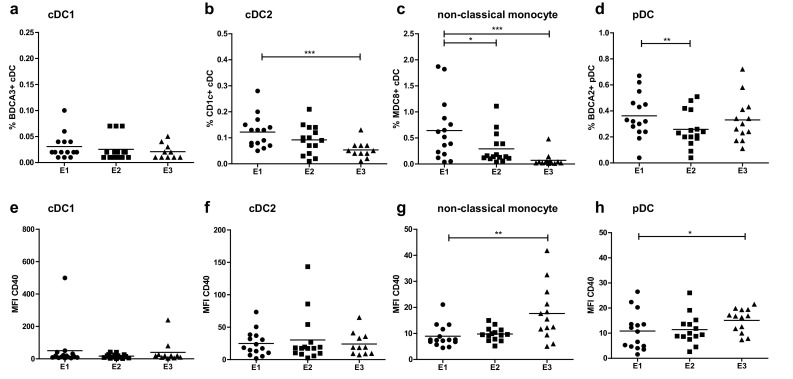
Frequencies and median fluorescence index (MFI) of the activation marker CD40 on cDC1 (**a** and **e**), cDC2 (**b** and **f**), non-classical monocytes (**c** and **g**) and pDC (**d** and **h**) over treatment: baseline (E1), third vaccination (E2), and follow-up (7–14 weeks, E3)

Frequencies of cDC2, non-classical monocytes and pDC, but not of cDC1, decreased during treatment (Fig. 5a–d). Maximal and significant decreases of cDC2 and non-classical monocytes subsets were reached after three vaccinations and multiple injections of CpG-B and IFN-α at E3. Significant decreases in pDC frequencies were observed at E2, and were shown to be only transient as pDC levels were restored at E3 after the 3rd vaccination and repeated CpG-B and IFN-α injections. Decreases in non-classical monocytes and pDC frequencies were paralleled by increases in their activation status at E3, as indicated in Fig. 5g, h by significant up-regulation of CD40 expression. In line with this, decreases in rates of non-classical monocytes and pDC at E2 were only observed in patients with CB, suggesting that indeed early decreases in frequencies of these antigen presenting cell subsets were associated with their activation and the patients’ response to treatment (See Supplementary Fig. 3a–d). Finally, after a transient non-significant increase at E2, mMDSC were significantly decreased at E3 in the CB group of patients (Supplementary Fig. 2e).

## Discussion

This phase II study in 15 patients with mRCC demonstrates that i.d. delivery of ATV with ATC/CpG-B/GM-CSF and systemic CpG-B/IFN-α is feasible, immunogenic and clinically active. All patients with CB developed a DTH response against ATC during the treatment and the induration area in the skin of patients with CB was significantly larger than in the patients with NCB, a difference that was not seen for KLH DTH suggesting an association of the DTH response against ATC with clinical activity as we have demonstrated before in stage III/IV melanoma patients [14]. We found relatively mild toxicity and no treatment-related deaths. Importantly, CpG-B as intradermally administered adjuvant (combined with GM-CSF) was shown to be safe and did not cause the ulcers that we observed in our previous studies where we used BCG instead [13, 39]. Unfortunately, we were not able to complete the pre-calculated enrollment level of 41 patients due to the approval of sunitinib as standard treatment for the patient group with mRCC. This has left us unable to perform a reliable assessment of the response rate (which was one of our primary objective) and overall survival. Nevertheless, a response rate of 20% (3/15 patients) was reached in this small cohort which is in line with our expectations prior to the start of the trial.

We found that ATC-specific IFN-ɣ production before ATV was related to clinical outcome (Fig. 3b). Interestingly, this difference in ATC responsiveness between patients with CB and patients with NCB was no longer detectable in the peripheral blood at E2 due to equally low IFN-ɣ levels in both patient groups, but reappeared at E3 with no detectable response to ATC in the patients with NCB. This may be due to a previously described phenomenon where ATC-specific T cells, upon their activation in the circulation, acquire the ability to migrate to the effector sites (the vaccination and tumor sites) under the influence of the immunotherapy and, thus, are transiently less abundant in the peripheral blood [40, 41]. This is corroborated by the relative (increase in) size of DTH to ATC at E2, which was elevated over E1 and significantly higher in patients with CB, further supporting the presence of an anti-tumor response that could localize to the site of tumor cell presence in the patients who responded to ATV administration (Fig. 3a). Our data thus suggest that ATC-specific IFN-ɣ production *in vitro* before treatment may be a predictive biomarker for treatment response, whereas the size of the DTH to ATC may be a first indicator of effective immunization against ATC (and treatment response) as early as 2 weeks after the first ATV.

Further immune monitoring revealed a decrease over treatment in cDC2, non-classical monocytes and pDC frequencies, which may also reflect recruitment of these myeloid effector subsets to effector sites. The frequencies of pDC (which are directly targeted by CpG-B via TLR-9) returned to baseline levels at E3, whereas cDC2 and non-classical monocytes frequencies further declined. Interestingly, the decline in non-classical monocytes and pDC frequencies was associated with a significant increase in their activation status (measured by CD40) at E3 which may be attributed to the bi-weekly CpG-B injections that started 1 week after E2. Also, mMDSC frequencies were significantly decreased by E3 in patients with CB.

This study shows that i.d. administration of ATV has clinical activity in a subset of patients but it may be even more interesting to look at possible combinations of this vaccination approach with other treatment modalities for mRCC. It was found for example that sunitinib has the ability to modulate the anti-tumor immune response by reversing MDSC accumulation and Treg elevation in RCC [42]. In a randomized study in metastatic renal cell carcinoma, it was demonstrated that the combined treatment with avelumab (anti-PDL1) and axitinib resulted in an improved progression-free survival as compared to sunitinib alone, suggesting that tyrosine kinase inhibitors have at least an added effect to immunotherapy (Abstract ESMO LBA6_PR ‘JAVELIN Renal 101). Therefore, when combined with therapeutic vaccines, sunitinib may help to overcome tumor-induced immune escape leading to increased numbers of tumor-infiltrating lymphocytes and tumor-specific CD8^+^ T cells, as well as enhanced tumor eradication and improved survival, as was previously shown in murine models [43, 44]. In patients, however, a recent randomized controlled phase III trial showed that there was no clinical benefit from the addition of a multipeptide cancer vaccine to sunitinib and even though T cell responses and monocyte counts were only assessed in a subset of the patients in the combination arm (which left the authors unable to compare these parameters between the combination and sunitinib monotherapy), the authors actually found evidence for a potential immune inhibitory role of sunitinib [45]. This seems to be in stark contrast with previous findings by us and others showing favorable immune modulation in patients with mRCC [35, 46], but may be due to differential effects of sunitinib in peripheral blood versus the tumor microenvironment [47]. As depicted in Fig. 4, we also observed an increase in the number of activated B cells (by CD86) and T cells (by CTLA-4 and PD-1). CTLA-4 and PD-1 are up-regulated when T cells become activated, which in the case of PD-1 has also been linked to neo-antigen specificity [48], and since the introduction of ICI, we know that selectively inhibiting these immune checkpoints can result in unprecedented anti-tumor activity. Moreover, it has become clear that response to ICI relies on T cells reactive to highly individualized neo-antigens [49]. In contrast to allogeneic or peptide-based vaccines, autologous vaccines cover all the personal (neo-) antigens that the tumor may express. In this light, ATV approaches may be able to enhance the sensitivity to ICI. Obstacles to further clinical development of ATV, however, are the fact that they do not qualify as a pharmaceutical product which makes funding of further development challenging, and the fact that vaccine production is laborious and dependent on the availability of tumor material. Nonetheless, 90% of the patients that were assessed for inclusion in this trial had enough tumor material available for successful vaccine production.

Patients received IFN-α after the third vaccination (E2) and since the clinical response evaluation (E3) was 12 weeks after the start of this active drug, it is possible that the clinical responses were (in part) the result of this treatment. Unfortunately we are unable to discriminate between the clinical effects of the ATV and the IFN-α in this trial. However, clinical responses in mRCC were previously demonstrated by others in vaccine-based clinical trials (without IFN-α) with cultured CD83 + blood DC loaded with autologous tumor cell lysates [50], DC pulsed with MUC1-derived peptides [51], a multipeptide cancer vaccine [52] and RNA coding for tumor-associated antigens [53]. As for the immunomonitoring data, all the findings obtained at E1 and E2 are the result of the ATV and that the findings at E3 can be attributed to either ATV or IFN-α, or both.

In conclusion, our data show that our ATV approach combined with IFN-α in mRCC is feasible, well tolerated and clinically active. Moreover, this treatment approach induced DTH responses against ATC and systemic activation of circulating PBDC and T cells in mRCC patients. In addition, preexisting ATC responsiveness of circulating T cells may be predictive for clinical outcome following treatment. Based on our observations in these 15 patients, further investigation of our ATV approach and current treatment modalities is indicated to improve response rates in this patient group.

## Electronic supplementary material

Below is the link to the electronic supplementary material.

Supplementary material 1 (PDF 276 KB)

## Abbreviations

ATC: Autologous tumor cells
ATV: Autologous tumor cell vaccine
CB: Clinical benefit
cDC: Conventional dendritic cells
CpG-B: Class-B CpG oligodeoxynucleotide
CpG ODN: Cytosine-phosphate-guanine oligodeoxynucleotides
CR: Complete response
CT: Computed tomography
DTH: Delayed type hypersensitivity
E (1,2,3): Evaluation (1,2,3)
ICI: Immune checkpoint inhibitors
KLH: Keyhole limpet hemocyanine
med. fl.: Median fluorescence
MFI: Median fluorescence index
mMDSC: Monocytoid myeloid-derived suppressor cells
mRCC: Metastatic renal cell carcinoma
NCB: No clinical benefit
PBDC: Peripheral blood dendritic cells
PD: Progressive disease
pDC: Plasmacytoid dendritic cells
PR: Partial response
SD: Stable disease
Tcm: Central-memory T cells
Teff: Effector T cells
Tem: Effector-memory T cells
Tn: Naïve T cells
UMC: University medical center
VU: Vrije Universiteit

## References

[1] Yagoda A, Bander NH (1989). Failure of cytotoxic chemotherapy, 1983–1988, and the emerging role of monoclonal antibodies for renal cancer. Urol Int.

[2] Coppin C, Porzsolt F, Autenrieth M, Coppin C (2004). Immunotherapy for advanced renal cell cancer. Cochrane database of systematic reviews.

[3] Rodriguez-Vida A, Hutson TE, Bellmunt J, Strijbos MH (2017). New treatment options for metastatic renal cell carcinoma. ESMO Open.

[4] Motzer RJ, Tannir NM, McDermott DF (2018). Nivolumab plus Ipilimumab versus sunitinib in advanced renal-cell carcinoma. N Engl J Med.

[5] Schumacher TN, Schreiber RD (2015). Neoantigens in cancer immunotherapy. Science.

[6] Keenan BP, Jaffee EM (2012). Whole cell vaccines–past progress and future strategies. Semin Oncol.

[7] McCune CS, O’Donnell RW, Marquis DM, Sahasrabudhe DM (1990). Renal cell carcinoma treated by vaccines for active specific immunotherapy: correlation of survival with skin testing by autologous tumor cells. Cancer Immunol Immunother.

[8] Jocham D, Richter A, Hoffmann L (2004). Adjuvant autologous renal tumour cell vaccine and risk of tumour progression in patients with renal-cell carcinoma after radical nephrectomy: phase III, randomised controlled trial. Lancet.

[9] May M, Brookman-May S, Hoschke B (2010). 10-year survival analysis for renal carcinoma patients treated with an autologous tumour lysate vaccine in an adjuvant setting. Cancer Immunol Immunother.

[10] Hoover HC, Brandhorst JS, Peters LC (1993). Adjuvant active specific immunotherapy for human colorectal cancer: 6.5-year median follow-up of a phase III prospectively randomized trial. J Clin Oncol.

[11] Berd D, Sato T, Maguire HC (2004). Immunopharmacologic analysis of an autologous, hapten-modified human melanoma vaccine. J Clin Oncol.

[12] Simons JW, Mikhak B, Chang JF (1999). Induction of immunity to prostate cancer antigens: results of a clinical trial of vaccination with irradiated autologous prostate tumor cells engineered to secrete granulocyte-macrophage colony-stimulating factor using ex vivo gene transfer. Cancer Res.

[13] Vermorken JB, Claessen AM, van Tinteren H (1999). Active specific immunotherapy for stage II and stage III human colon cancer: a randomised trial. Lancet.

[14] Baars A, Claessen AM, van den Eertwegh AJ (2000). Skin tests predict survival after autologous tumor cell vaccination in metastatic melanoma: experience in 81 patients. Ann Oncol Off J Eur Soc Med Oncol.

[15] Baars A, Claessen AME, Wagstaff J (2002). A phase II study of active specific immunotherapy and 5-FU/Leucovorin as adjuvant therapy for stage III colon carcinoma. Br J Cancer.

[16] Krieg AM (2012). CpG still rocks! update on an accidental drug. Nucleic Acid Ther.

[17] Krieg AM (2008). Toll-like receptor 9 (TLR9) agonists in the treatment of cancer. Oncogene.

[18] Halperin SA, Van Nest G, Smith B (2003). A phase I study of the safety and immunogenicity of recombinant hepatitis B surface antigen co-administered with an immunostimulatory phosphorothioate oligonucleotide adjuvant. Vaccine.

[19] Ellis RD, Martin LB, Shaffer D (2010). Phase 1 trial of the plasmodium falciparum blood stage vaccine MSP142-C1/Alhydrogel with and without CPG 7909 in malaria naïve adults. PLoS One.

[20] Speiser DE, Liénard D, Rufer N (2005). Rapid and strong human CD8^+^ T cell responses to vaccination with peptide, IFA, and CpG oligodeoxynucleotide 7909. J Clin Invest.

[21] Speiser DE, Schwarz K, Baumgaertner P (2010). Memory and effector CD8 T-cell responses after nanoparticle vaccination of melanoma patients. J Immunother.

[22] Karbach J, Gnjatic S, Bender A (2009). Tumor-reactive CD8^+^ T-cell responses after vaccination with NY-ESO-1 peptide, CpG 7909 and Montanide® ISA-51: Association with survival. Int J Cancer.

[23] Karbach J, Neumann A, Atmaca A (2011). Efficient in vivo priming by vaccination with recombinant NY-ESO-1 protein and CpG in antigen naive prostate cancer patients. Clin Cancer Res.

[24] Peters LC, Brandhorst JS, Hanna MG (1979). Preparation of immunotherapeutic autologous tumor cell vaccines from solid tumors. Cancer Res.

[25] Molenkamp BG, Sluijter BJR, van Leeuwen PAM (2008). Local administration of PF-3512676 CpG-B instigates tumor-specific CD8^+^ T-cell reactivity in melanoma patients. Clin Cancer Res.

[26] Sallusto F, Lenig D, Förster R (1999). Two subsets of memory T lymphocytes with distinct homing potentials and effector functions. Nature.

[27] Allan SE, Crome SQ, Crellin NK (2007). Activation-induced FOXP3 in human T effector cells does not suppress proliferation or cytokine production. Int Immunol.

[28] Wang J, Ioan-Facsinay A, van†„der†„Voort EIH (2007). Transient expression of FOXP3 in human activated nonregulatory CD4^+^ T cells. Eur J Immunol.

[29] Gavin MA, Torgerson TR, Houston E (2006). Single-cell analysis of normal and FOXP3-mutant human T cells: FOXP3 expression without regulatory T cell development. Proc Natl Acad Sci USA.

[30] Huijts CM, Santegoets SJ, de Jong TD (2017). Immunological effects of everolimus in patients with metastatic renal cell cancer. Int J Immunopathol Pharmacol.

[31] Dzionek A (2001). BDCA-2, a Novel plasmacytoid dendritic cell-specific type II C-type lectin, mediates antigen capture and is a potent inhibitor of interferon alpha/beta induction. J Exp Med.

[32] van Leeuwen-Kerkhoff N, Lundberg K, Westers TM (2017). Transcriptional profiling reveals functional dichotomy between human slan + non-classical monocytes and myeloid dendritic cells. J Leukoc Biol.

[33] Schäkel K, Mayer E, Federle (1998). A novel dendritic cell population in human blood: one-step immunomagnetic isolation by a specific mAb (M-DC8) and in vitro priming of cytotoxic T lymphocytes. Eur J Immunol.

[34] Schäkel K, Kannagi R, Kniep B (2002). 6-Sulfo LacNAc, a novel carbohydrate modification of PSGL-1, defines an inflammatory type of human dendritic cells. Immunity.

[35] van Cruijsen H, van der Veldt AAM, Vroling L (2008). Sunitinib-induced myeloid lineage redistribution in renal cell cancer patients: CD1c + dendritic cell frequency predicts progression-free survival. Clin Cancer Res.

[36] Filipazzi P, Valenti R, Huber V (2007). Identification of a new subset of myeloid suppressor cells in peripheral blood of melanoma patients with modulation by a granulocyte-macrophage colony-stimulation factor–based antitumor vaccine. J Clin Oncol.

[37] Santegoets SJ, Stam AG, Lougheed SM (2014). Myeloid derived suppressor and dendritic cell subsets are related to clinical outcome in prostate cancer patients treated with prostate GVAX and ipilimumab. J Immunother cancer.

[38] Sokal JE (1975). Measurement of delayed skin-test responses. N Engl J Med.

[39] Baars A, Claessen A, Wagstaff J (2002). A phase II study of active specific immunotherapy and 5-FU/Leucovorin as adjuvant therapy for stage III colon carcinoma. Br J Cancer.

[40] Harris RC, Chianese-Bullock KA, Petroni GR (2012). The vaccine-site microenvironment induced by injection of incomplete freundʼs adjuvant, with or without melanoma peptides. J Immunother.

[41] Hailemichael Y, Dai Z, Jaffarzad N (2013). Persistent antigen at vaccination sites induces tumor-specific CD8 + T cell sequestration, dysfunction and deletion. Nat Med.

[42] Ko JS, Zea AH, Rini BI (2009). Sunitinib mediates reversal of myeloid-derived suppressor cell accumulation in renal cell carcinoma patients. Clin Cancer Res.

[43] Draghiciu O, Nijman HW, Hoogeboom BN (2015). Sunitinib depletes myeloid-derived suppressor cells and synergizes with a cancer vaccine to enhance antigen-specific immune responses and tumor eradication. Oncoimmunology.

[44] Farsaci B, Donahue RN, Coplin MA (2014). Immune consequences of decreasing tumor vasculature with antiangiogenic tyrosine kinase inhibitors in combination with therapeutic vaccines. Cancer Immunol Res.

[45] Rini BI, Stenzl A, Zdrojowy R (2016). IMA901, a multipeptide cancer vaccine, plus sunitinib versus sunitinib alone, as first-line therapy for advanced or metastatic renal cell carcinoma (IMPRINT): a multicentre, open-label, randomised, controlled, phase 3 trial. Lancet Oncol.

[46] Finke JH, Rini B, Ireland J (2008). Sunitinib reverses type-1 immune suppression and decreases T-regulatory cells in renal cell carcinoma patients. Clin Cancer Res.

[47] Ko JS, Rayman P, Ireland J (2010). Direct and differential suppression of myeloid-derived suppressor cell subsets by sunitinib is compartmentally constrained. Cancer Res.

[48] Gros A, Parkhurst MR, Tran E (2016). Prospective identification of neoantigen-specific lymphocytes in the peripheral blood of melanoma patients. Nat Med.

[49] McGranahan N, Furness AJS, Rosenthal R (2016). Clonal neoantigens elicit T cell immunoreactivity and sensitivity to immune checkpoint blockade. Science.

[50] Höltl L, Rieser C, Papesh C (1998). CD83 + blood dendritic cells as a vaccine for immunotherapy of metastatic renal-cell cancer. Lancet.

[51] Wierecky J, Müller MR, Wirths S (2006). Immunologic and clinical responses after vaccinations with peptide-pulsed dendritic cells in metastatic renal cancer patients. Cancer Res.

[52] Walter S, Weinschenk T, Stenzl A (2012). Multipeptide immune response to cancer vaccine IMA901 after single-dose cyclophosphamide associates with longer patient survival. Nat Med.

[53] Rittig SM, Haentschel M, Weimer KJ (2011). Intradermal vaccinations with RNA coding for TAA generate CD8 + and CD4 + immune responses and induce clinical benefit in vaccinated patients. Mol Ther.

