# Supplementary material for: Autologous tumor cell vaccination combined with systemic CpG-B and IFN-α promotes immune activation and induces clinical responses in patients with metastatic renal cell carcinoma: a phase II trial
Source: Cancer Immunol Immunother. 2019 Mar 9;68(6):1025–35. doi: 10.1007/s00262-019-02320-0 (PMC6529601; doi:10.1007/s00262-019-02320-0)
Supplement: Supplementary file 1 — Supplementary material 1 (PDF 276 KB) [file 262_2019_2320_MOESM1_ESM.pdf]

**Supplemental table 1. Adverse events**

| Adverse Event | Any grade | Grade 1 | Grade 2 | Grade 3 or 4 |
|---|---|---|---|---|
| Fatigue | 13 | 7 | 5 | 1 |
| Fever | 10 | 10 | | |
| GGT elevation | 10 | 3 | 1 | 6 |
| Anemia | 8 | 1 | 5 | 2 |
| ALP elevation | 6 | | 3 | 3 |
| Anorexia | 6 | 5 | 1 | |
| Malaise | 6 | 6 | | |
| Night sweats | 6 | 5 | 1 | |
| Skin rash | 6 | 6 | | |
| Chills | 5 | 5 | | |
| Creatinine elevation | 5 | 5 | | |
| Itch | 5 | 5 | | |
| Pain | 5 | 5 | | |
| Cough | 4 | 4 | | |
| Dizziness | 4 | 3 | | 1 |
| Nausea | 4 | 4 | | |
| Myalgia | 3 | 3 | | |
| Abdominal pain | 2 | 2 | | |
| Constipation | 2 | 2 | | |
| Aphasia | 1 | | | 1 |
| Attention disorder | 1 | 1 | | |
| Chest pain | 1 | 1 | | |
| Diarrhea | 1 | 1 | | |
| Disturbed balance | 1 | | | 1 |
| Dry skin | 1 | 1 | | |
| Dyspnea | 1 | | 1 | |
| Hemoptysis | 1 | 1 | | |
| Headache | 1 | 1 | | |
| Hearing loss | 1 | 1 | | |
| Hemiplegia | 1 | | | 1 |
| Hypertension | 1 | 1 | | |
| Insomnia | 1 | 1 | | |
| Melena | 1 | | | 1 |
| Nycturia | 1 | 1 | | |
| Edema | 1 | | 1 | |
| Pancreatitis | 1 | | 1 | |
| Rectal blood loss | 1 | 1 | | |
| Rhinitis | 1 | 1 | | |
| Sensory Neuropathy | 1 | | | 1 |
| Vomiting | 1 | 1 | | |

Abbreviations: GGT, gamma-glutamyl transferase; ALP, Alkaline phosphatase

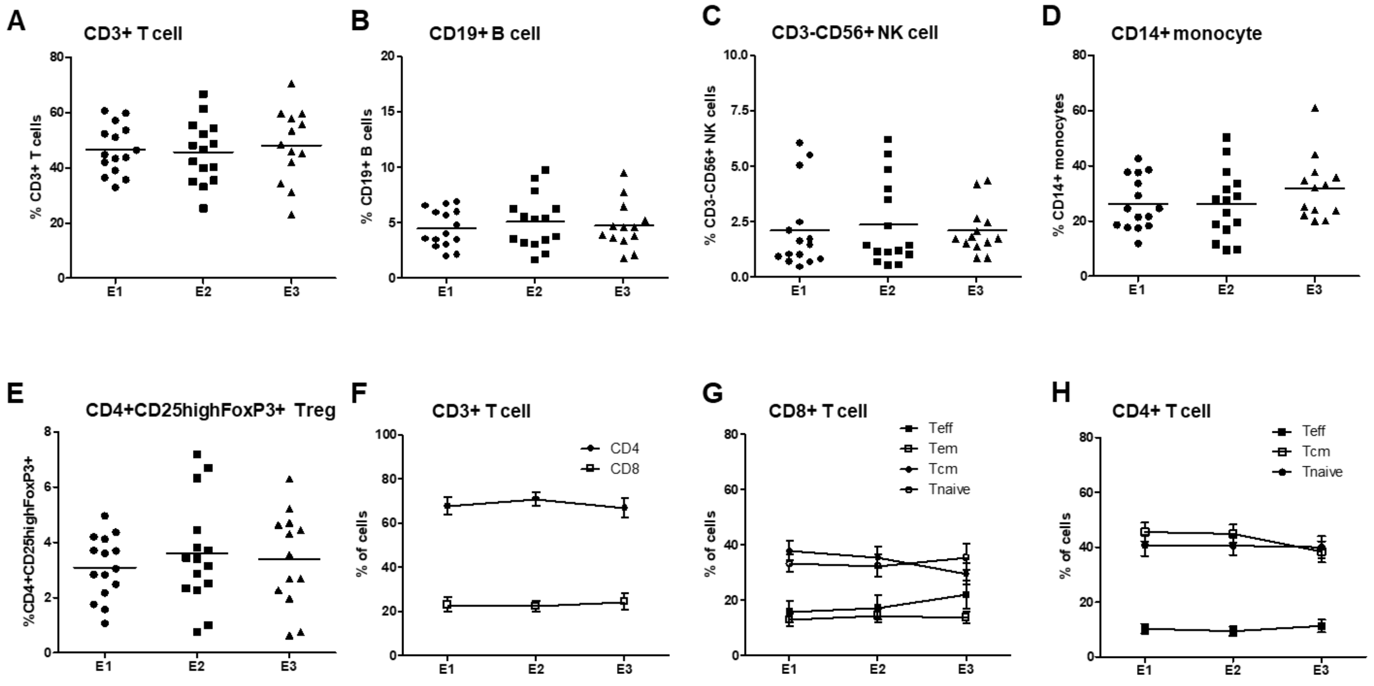


**Supplemental figure 1** Frequencies of circulating $CD3^+$ T cells (A), $CD19^+$ B cells (B), $CD3^-CD56^+$ NK cells (C), $CD14^+$ classical monocytes (D) and $CD4^+CD25^{hi}FoxP3^+$ Tregs (E) and frequencies of circulating $CD4^+$ T cells, $CD8^+$ T cells (F) and $CD4^+$ (G) or $CD8^+$ (H) Tn, Teff, Tcm or Tem cells at baseline (E1), third vaccination (E2), and follow-up (7-14 weeks, E3).

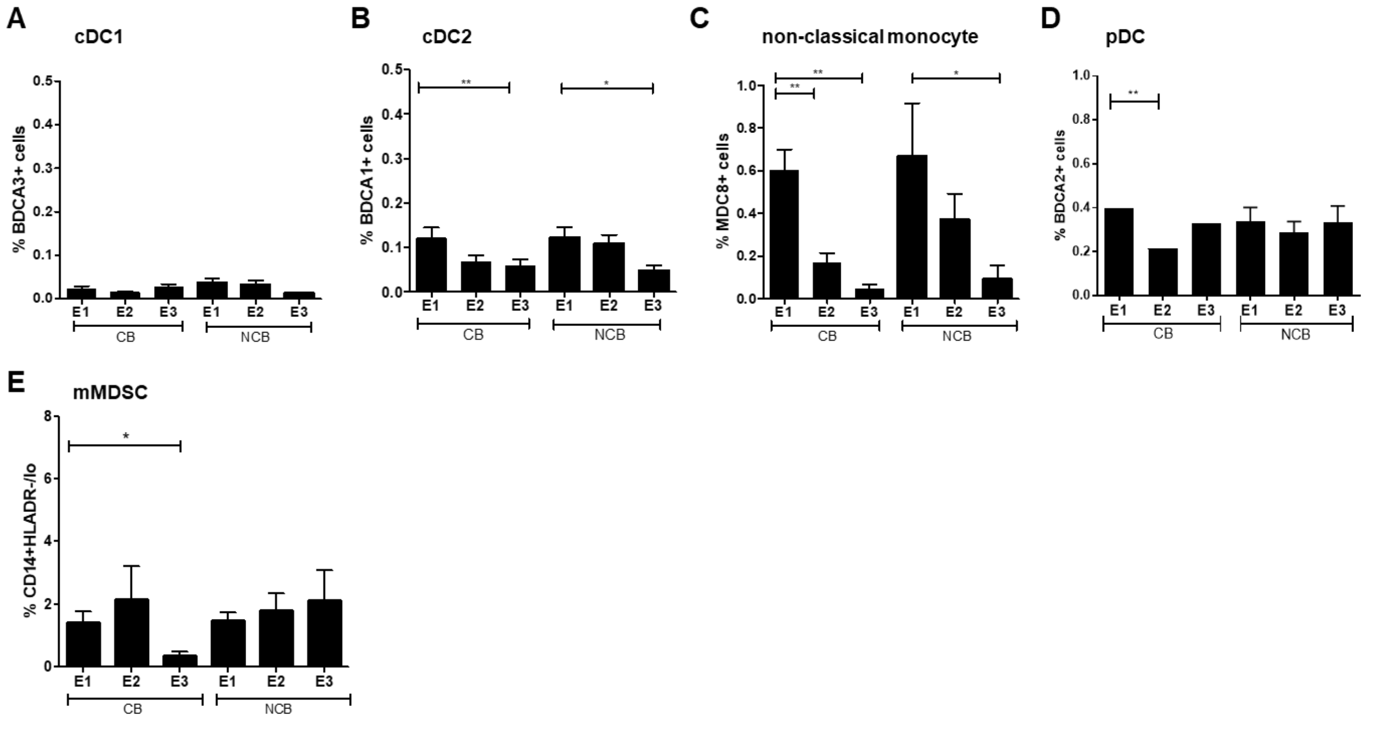


**Supplemental figure 2** Comparative analysis of PBDC frequencies between patients with clinical benefit (CB: stable disease [SD], partial response [PR] and complete response [CR]) and patients with no clinical benefit (NCB: progressive disease [PD]).at baseline (E1), third vaccination (E2), and follow-up (7-14 weeks, E3).
